# Reconciling the definitions of raw meat-based diets and biologically appropriate raw foods for companion animals: a mini review

**DOI:** 10.3389/fvets.2025.1547953

**Published:** 2025-07-08

**Authors:** Samuel S. Kiprotich, Eric Altom, Robert Mason, Charles G. Aldrich

**Affiliations:** ^1^Department of Grain Science and Industry, Kansas State University, Manhattan, KS, United States; ^2^Animal Nutrition and Health Division, Balchem Corporation, Montvale, NJ, United States

**Keywords:** raw pet food, companion animals, raw meat based diets, RMBDs, biologically appropriate raw foods, BARFs, cats, dogs

## Abstract

There is an increasing demand for pet foods considered “natural,” raw, uncooked, minimally processed, and those free of synthetic preservatives used to inactivate spoilage and pathogenic microorganisms. These diets are referred to as raw meat-based diets (RMBDs), biologically appropriate raw foods (BARFs) or raw animal products (RAPs). However, the definitions of these diets are highly subjective and rely on the interpretation of pet food manufacturers, researchers, consumers, and animal food regulatory authorities. The lack of standardized definitions hampers the necessary progress in research required to better understand this rapidly growing segment of pet food. The different definitions reduce the efficiency of international and interstate commerce between pet food manufacturers, ingredient suppliers, consumers and the regulatory authorities in different geographical jurisdictions. There is a plethora of existing literature defining and describing what raw pet foods are. Thus, a comprehensive search for published research was conducted regarding definitions and word descriptions tangential to these raw pet foods. This mini review paper explored multiple research and review articles that attempted to define “raw pet foods,” and the word descriptions they used. This review focuses on RMBDs, BARFs, “raw pet foods,” and RAPs as defined from an academic, processing, regulatory and consumer perspective. Furthermore, we have proposed a new working definition for these diets as “Raw and Minimally Processed” (RAMP) pet food to reflect consumer, regulatory and academic needs, and expectations. Reconciling these definitions will lay a better framework for communication, research, regulation, and commerce between stakeholders in the pet food industry.

## Introduction

There is an increasing demand for minimally processed pet foods such as raw meat-based diets (RMBDs) or biologically appropriate raw foods (BARFs) or raw animal products (RAPs) in developed countries around the world. This phenomenon largely has been attributed to anthropomorphism whereby companion animals are treated as family members (1–3). Thus, the dietary changes currently being observed in the human population where more people opt for healthier and “natural” food choices can be correlated to the dietary choices pet parents make for their animals (4).

Raw meat-based diets (RMBDs) are categorized as a subset of minimally processed commercial diets (MPCD) or minimally processed home prepared diets (MPHD) for companion animals (2, 5). The categorization of these diets into commercial and home prepared RMBDs is necessary because these diets are manufactured and retailed under different regulatory jurisdictions. These diets consist of mainly uncooked ingredients (lamb, pork, poultry, beef, venison, organ meats or offal, eggs, shells, or diary) which may be supplemented with tubers, vegetables, fruits, or cereals and fed to dogs and cats (2, 6). In contrast, BARFs were originally defined as “bones and raw food” or “raw animal products,” (RAPs) but are now euphemistically referred to as biologically appropriate raw foods. This concept was first promoted by an Australian veterinarian and nutritionist, Ian Billinghurst (6) and Ankers (17). The aim of BARF is to mimic diets like those of the dogs’ wolf ancestor which consume prey high in proteins (raw meats, bones, organs) and low in carbohydrates. From these descriptions, RMBDs and BARFs seem like similar diets, though Freeman et al. (6) distinguished these diets according to the motivations behind their formulation and preparation. Freeman et al. (6) reported that commercial RMBDs are formulated to meet nutrient requirements set forth and enforced by Association of American Feed Control Officials (AAFCO) and their affiliated state feed control officials. The BARFs on the other hand are formulated to mimic wolf diets, ingredients or recipes that vary compositionally and are mostly prepared at home. Though an overlap of the two concepts is unavoidable.

The definitions and descriptions for BARF, RAPs and/or RMBDs continue to muddy the debates surrounding these somewhat controversial pet diets. This is further exacerbated by the fact that these diets are raw, uncooked, or unpasteurized and could be vectors of foodborne bacteria (2). Therefore, reconciling the definitions of these diets might allow for research into ingredient formulations and pathogen mitigation approaches to be streamlined. Minimum processing platforms such as fermentation, use of food acidulants, or freeze-drying would help improve the quality and shelf-life of these diets. This mini review will focus on RMBDs, RAPs and BARFs manufactured and retailed commercially. The objective is to reconcile the definitions of RMBDs, RAPs and BARFs from an academic, processing, regulatory and consumer perspective with the overarching goal of improving scientific and applied communication encompassing manufacturing, pathogen mitigation approaches, and quality of raw pet food for companion animals. The secondary objective is to propose a new working title that can be used to describe raw pet food.

## Methodology

There is limited published research investigating the nomenclature and semantics used to describe commercial raw pet food as most research focuses on mitigating enteric foodborne pathogens inherently found in these diets. Thus, to understand the definitions of raw pet food, we conducted a systematic search of the literature. The search was conducted by selecting key words, which were search variables in selected databases, and then the inclusion/exclusion criterion was established. The key words included “raw dog food,” “dog,” “cat,” “RMBD,” “raw meat-based diet,” “raw pet food,” “raw animal products,” “RAP,” “BARF,” and “biologically appropriate raw food.” These key words were applied to Google Scholar and Scopus with no limit to years or language. Original research and review articles with clear definitions of BARF, RMBDs or RAPs were considered for this mini review. Articles in book chapters, patents, trade publications, extension bulletins, and conference abstracts were excluded from this section. Articles that followed AAFCO guidelines in their definitions or formulations or were intended for the North American pet food market were included in the search while those under European jurisdiction and guidelines stipulated by European Pet Food Industry Federation (FEDIAF) were excluded because they had different definitions of raw pet food that do not apply in North America.

### Semantics of raw pet food

Most companion animals are fed commercially produced dry, extruded kibbles or wet canned foods and thus feeding animals with RMBDs, BARFs, or RAPs is less conventional and somewhat controversial as these diets contain uncooked skeletal muscle, fat, internal organs, cartilage, and bones from farm animals (ruminants, pigs, and poultry), horses, game, and fish (6–9).

### Academic and scholarly definitions of raw pet food

Previous researchers in the discipline (10) described BARFs as homemade complete raw food diets, with most popular recipes consisting of 60% raw, meaty bones with the rest of the ration comprising “a wide variety of foods that a wild dog would eat.” These other ingredients would include vegetables to mimic the stomach contents of prey, offal, dairy, or eggs, although each meal was not necessarily nutritionally balanced (11). Another raw food diet for companion animals that loosely falls under the BARF category would be the ultimate diet program (UDP) described by Kymythy Schultze in 1998. The UDP food pyramid consists of the largest proportion of the diet as raw meat (muscles and organs), bones, raw eggs and supplemented with small quantities of raw vegetables (12). Buff et al. (13) described forms of raw pet foods that are instinctual or ancestral diets and are intended to feed pets according to their physiological metabolism and preferences, rather than just meeting the physiological nutritional needs of the animal. Buff et al. (13) defined instinctual diets as feeding pets according to their innate preferences with the assumption that pets choose foods that meet their nutritional requirements whereas ancestral diets are based on the philosophy of feeding animals’ diets similar to that of their evolutionary ancestors with the assumption that these foods will meet the physiological and metabolic needs of the companion animal. Despite differing philosophies, the ancestral and instinctual diets are “supposedly” higher in proteins and lower in carbohydrates and thus are assumed to be healthier alternatives to commercial diets because of their smorgasbord presentation. However, the nutritional adequacy and “health benefits” of home-prepared ancestral and instinctual diets have been questioned by researchers. For instance, Villaverde and Chandler (14) reported a lack of evidence that feeding domestic cats with home-made diets resulted in longer and healthier lives than when they were fed commercial diets. There are also instances when home-prepared home diets are poorly formulated, resulting in inadequate essential nutrients, thus making them nutritionally inferior to commercial diets (14). In summary, there are no regulatory definitions for the ancestral and instinctual diets, thus there are no commercial diets that fall under this category. Nonetheless, we can hypothesize that these diets are minimally cooked or served raw to these pets if they are to reflect their philosophical origins.

### Regulatory bodies definition of raw pet food

In contrast to the prevailing folklore, AAFCO (18) the primary body for language regarding labeling pet foods in North America and other countries that import American made pet foods has several terms defined that must be considered. For example, “raw” is defined as a food in its natural state not having been subjected to heat during its preparation. This would eliminate pet foods that have been heat-processed captured within the definition of RMBDs, RAPs, or BARFs. However, AAFCO’s definition of “raw” excludes the utilization of non-thermal processing technologies such as high-pressure, food acidulants and fermentation that can be used to control pathogen mitigation and enhance the safety of these diets. These are clearly processing steps. Moreover, raw pet foods stipulated as “fresh” by AAFCO cannot be subjected to freezing, treatment by cooking, drying, rendering, hydrolysis, addition of salt, curing agents, natural or synthetic preservatives, other processing aids or preservation by means other than refrigeration. Thus, based on this information, a working definition of raw pet food might be meat, tissues, organs, bones that can be combined with cereals and vegetables and fed to companion animals in their uncooked/unheated form. Additionally, food processing aids such as organic acidulants, high-pressure processing, fermentation, hydrolyzation, or irradiation could be used as mitigation approaches for foodborne pathogens in these diets.

### Consumer definitions of raw pet food

Raw pet food from a consumer perspective depends on their own personal relationship with food. What these consumers characterize as food and what it symbolizes in their lives is a great predictor of their perspective toward raw diets (15). Furthermore, consumers that consumed in their own diet “natural,” “wholesome,” organic or raw and minimally processed foods gravitate toward unconventional pet foods such as home-prepared diets, “natural,” organic, human food grade ingredients, and raw animal products (15). In some respects, the proponents and consumers of raw pet food take on the persona of a “movement.” A survey conducted by Morelli et al. (16) sought to understand owners’ motivations, practices, and attitudes toward raw meat-based diets for dogs. They reported that over half of their respondents had abandoned commercial pet food (dry and wet foods) for RMBDs as they considered the latter to be more “natural” and healthier. Also, 80% of their respondents that had abandoned commercial pet diets showed remarkable distrust toward the lack of clarity (ambiguity) on pet food ingredient labels. Moreover, 57% of their correspondents believed that switching to RMBDs allowed them to control the quality and composition of ingredients that were fed to their pets. This translates into the motivation for some pet owners to prepare raw pet food for their animals at home (2). The recurring theme as we attempt to understand consumer perspectives of RMBDs is that they prefer their animals to eat “natural,” “wholesome,” organic, or human grade food which is minimally processed with succinct lists of ingredients and food processing aids.

## Discussion

There is an impasse looming in the near future as the demand for these raw pet foods continues to increase. This impasse, in our opinion, is going to pit pet owners versus academic and regulatory authorities who provide research and guidance to pet food manufacturers. Pet parents want “natural” and minimally processed pet foods which are perceived to be healthier, while manufacturers and regulators are concerned with the safety of these RMBDs because regulatory definitions disqualify thermal treatment for pathogen inactivation. Other avenues or technology platforms may prove effective, such as HPP or the application of food acidulants. Though for these techniques, investigation of the efficacy and application strategies are in their infancy stages as antimicrobial intervention platforms.

We hypothesize that the clash between pet owners and manufacturers is going to arise from regulatory language and what quality of ingredients are going to be available, and the food processing platforms that are utilized. If not managed effectively, a breakdown in communication between all the concerned stakeholders might result in consumer distrust. This might arise because of consumers’ little or lack of general understanding of statutory and regulatory definitions on ingredients and processing platforms as this has a direct impact on commercial pet foods. For instance, AAFCO descriptive terms such as “organic,” “fresh,” or “raw” might not meet consumer expectations when they purchase raw pet food due to their misinterpretation of these terms. The conundrum here is that these terms from a food processing, regulatory and scholarly perspective have different semantics from what consumers expect. For instance, AAFCO defines “natural” as ingredients that are in their unprocessed state whereas “raw” would refer to ingredients that have not been exposed to heat in any form.

On the other hand, AAFCO gives manufacturers much more leeway to help them find means to enhance the safety of their diets using non-thermal approaches for pathogen mitigation and food additives. From an academic and regulatory standpoint, the definition of “raw” by AAFCO rationale is challenging because these diets are notorious vehicles for the transmission of foodborne pathogens if not adequately pasteurized (4). However, most pet owners that buy raw diets do so because of the belief that they are healthier and “natural,” and would expect product descriptions and labeling to meet these expectations and animal needs. The motivations of raw pet food customers are based on the health of their animals, especially if the same customers believe that less processed and raw human foods are better and healthier than ultra-processed pet food ingredients and by-products. Thus, calling these diets “raw” would be adequate and would allow for the utilization of multiple non-thermal pathogen mitigation approaches, but this increases the list of ingredients on a label which might not meet the needs and expectations of consumers.

## Way forward

The reconciliation of the definitions of raw pet food is going to be necessary if consumer expectations are to be met without compromising the microbial safety of these diets. The current description of raw diets includes RAPs, RMBDs or BARFs whose academic definitions are focused on nutrition and safety but are open to interpretation from a regulatory standpoint. Therefore, we are proposing a new academic description of raw pet food that attempts to meet consumer needs while falling under the correct processing and regulatory guidelines.

New terminology for raw pet food may be needed. As a strawman, the following is offered to begin the discussion: “Raw and Minimally Processed” (RAMP) pet foods. Our thought process hinges on the definition of “raw” set forth by AAFCO which gives us leeway to utilize a plethora of non-thermal pathogen mitigation approaches and platforms without violating regulations enforced by feed control officials. The “minimally processed” section of the terminology borrows from the description first proposed Raditic (5). We also believe that adding the description of “minimally processed” to the description of these diets helps set the expectations of the consumer because this allows manufacturers the opportunity to use processing aids such as high-pressure pasteurization, irradiation, freezing or food additives such as food acidulants. Our overarching goal is to succinctly communicate to an already distrustful consumer base that the minimal processing that these diets undergo is to ensure their microbial safety and nutrition, and thus limiting the backlash of having what consumers often describe as unpopular, “long” and “unclear” ingredient lists.

## Conclusion

The era of raw and minimally processed (RAMP) pet foods is upon us. It is in our common interests (public health safety) to ensure that these diets are safe, nutritious, and meet regulatory requirements without sacrificing consumer expectations and needs. Therefore, as additional research in safety and nutrition in the RAMP segment of pet food increases, the nomenclature and semantics that describe these diets should not only be reconciled to meet the requirements of manufacturers and state feed control authorities, but also consumer expectations and demands. With consumer expectations and state feed control officials’ requirements fulfilled, pet food manufacturers can then proceed efficiently to provide nutritious and microbially safe RAMP diets to meet the increasing demands of consumers.
